# Alterations in the penile artery blood flow hemodynamics and hormonal profile in jacks *(Equus asinus*) after a single administration of human chorionic gonadotropin (hCG)

**DOI:** 10.1186/s12917-025-05025-y

**Published:** 2025-10-03

**Authors:** Amr F. El karmoty, Mohamed Fathi, Ibrahim A. Emam, Ayman Tolba, Elshymaa A. Abdelnaby, Abdulrahman K. Alhaider, Yara S. Abouelela

**Affiliations:** 1 Department of Anatomy and Embryology, Faculty of Veterinary Medicine, Cairo University, Giza Squarem, 12211 Egypt; 2 Department of Theriogenology, Faculty of Veterinary Medicine, Cairo University, Giza, Egypt; 3 Department of Surgery, Anesthesiology and Radiology, Faculty of Veterinary Medicine, Cairo University, Giza, 12211 Egypt; 4 Department of Clinical Sciences, College of Veterinary Medicine, King Faisal University, P.O. Box 400, 31982 Al‑Ahsa, Saudi Arabia

**Keywords:** Donkey, Penile artery, Erection, HCG, Anatomy, Doppler

## Abstract

The penis of the donkey is a musclocavernous type as the erection of the penis is dependent on the blood flow within the cavernous tissue inside the penis,This current study aimed for the first time to determine the effect of human chorionic gonadotropin (hCG) injection on penile artery blood flow hemodynamics and erection process after carefully studying the anatomical architecture of the penile blood supply. This study was conducted on 23 donkeys as 3 Male cadavers were used for anatomical findings and the other 20 live jacks were subdivided into two equal groups (Group I [treated jacks; *n* = 10] and Group II [control males; *n* = 10]). The arterial supply of the penis of the horse is oriented by external pudendal, internal pudendal and obturator arteries. The penile artery Doppler indices were declined (*P* < 0.05) in the hCG group after a single injection of hCG by 2,4 and reached the lowest level at 6 h. The penile blood flow volume reached the highest level 6 h after injection. The levels of both nitric oxide (NO), estradiol (E2), and follicle-stimulating hormone (FSH) were elevated, reaching the peak point after 6 h. of injection for E2 and NO and 4 h for FSH. There was a positive correlation between penile blood flow and both NO and E2 (NO with *r* = 0.66; *P* = 0.01, and E2 with r = 0.69; *P* = 0.01). In conclusion, the single administration of hCG improves the penile artery vascularity and hormonal levels at 6 h. post-injection, which positively affects penile function and the erection process in jacks *(Equus asinus*).

## Introduction

When the penis retracts into the sheath, the retractor muscle contracts; when it relaxes, the penis extends from the sheath [1, 2]. The penis grows by two times in length, thickness, and the glans by three to four times when an individual is erect. The urethral fossa, a little pocket at the distal end of the glans, is where the urethra opens [3]. Penis erection needs an increase in the penile blood flow with enlargement of the body sinusoids due to an increase in pressure inside the penis with relaxation of the retractor penis muscle [3, 4].

The uses and benefits of Doppler applicability in human and veterinary medicine have a significant impact, especially in reproductive cases, such as the assessment of testicular and penile vascularisation, which may be interrelated to many infertility complications [5]. In the human field, it is mostly used for perfect diagnosis of testicular torsion and varicocele**,** in addition to penile dysfunction as abnormal erection [6, 7]. As well as for the veterinary aspects, many studies reported the Doppler ultrasound of testes in equines [8], in dogs [9, 10] and camels [11].

Pozor [12], reported that the use of the Doppler accurately depends mainly on the knowledge of the anatomical picture of the arterial architecture, so the anatomy of the blood vessels and their ramifications is of great importance. The main source of the blood supply of the equine penis is the internal pudendal, obturator and cranial artery of the penis which is considered the continuation of the external pudendal artery [12, 13]. The anatomical description of the penial artery in the donkey was missed, so we focused on it on our results. The penile erection in donkeys is a psychoneuroendocrine mechanism combined with neurovascular stimulation manifested in blood engorgement of the sinusoidal spaces of both corpora cavernousum and spongiousum [14].

Human chorionic gonadotropin (hCG) is a chemical mediator created by trophoblast cells and eventually is considered as a part of the placenta, in animals and humans, hCG enhances testosterone release to perform its action on erection and is also used in the treatment of reduced libido and erectile dysfunction [15]**.** We aimed in this study to focus on the anatomical architecture of the penile blood supply and estimate the hemodynamic and hormonal variation under the effect of injection of a single dose of hCG on the penile erection of the adult jacks.

## Methodology

### Ethical approval

Our study was operated, including animal Manipulation and sampling according to the approval of the Veterinary Animal Care and Use Committee of the Faculty of Veterinary Medicine, Cairo University in strict compliance with the ARRIVE Guidelines 2.0 for reporting animal research, the National Institute of Health Guide for Care and Use of Laboratory Animals (NIH Publication no. 85–23, revised 2011) and the UK Animals (Scientific Procedures) Act 1986 with Approval number Vet CU 08072023724.

### Animal management, location and housing

The current study was performed in the Departments of Theriogenology and Anatomy and Embryology on 23 jacks (*n*=23; Equus asinus); at the Faculty of Veterinary Medicine, Cairo University (30.0154° N, 31.2103°) with an average temperature of 25° C during March 2023. 3 Male cadavers were used for anatomical findings, and the other 20 live jacks were subdivided into two equal groups with 8–10 years old, 4.3 ± 0.5 BCS, 670 ± 20 kg bodyweight (Group I [treated jackss; *n* = 10] and Group II [control males; *n* = 10]). All animals have normal cardiovascular systems and normal health conditions, and are normally vaccinated against some diseases. Therefore, this study was divided into two different experiments. Experiment 1: anatomy (*n*=3); and experiment 2, blood flow and blood sample collection (*n*=20).

All males were presented in indoor paddocks, and all were fed a commercial pelleted ration with free access to water all day. breeding soundness evaluation was performed on all males with an ultrasonographic evaluation on all genital organs, including testes, penis, and prepuce [16]. the penis and prepuce were examined firstly by palpation. 

### Collection of tissue samples

Three specimens of vascular anatomical architecture were collected from cadaver donkeys, which died in our clinic at the Faculty of Veterinary Medicine, Cairo University, Egypt, due to various causes, possibly accidents or during surgery, over a period of three months. The pelvic portions were transported to the laboratory in the Department of Anatomy and Embryology for assessment within 15 minutes after exsanguination.

### Vascular anatomical architecture

Three specimens were handled to examine the penial vascular anatomical architecture in adult jackss. The abdominal aorta was cannulated, after that, flushed thoroughly with saline to eliminate any blood clots, and then infused with 60% gum milk latex emulsion coloured red for arteries using ROTRING ink [17, 18]. Then the specimens were preserved in formalin 10% and 1% glycerine solution for 4 days aforementioned to manual dissection. Photographs were grabbed by a digital camera and edited by Photoshop CCx64 version. 

### Injection of human chronic gonadotropin (hcg)

The first group were injected with one dose of human chorionic gonadotropin (hCG; EPIFASI 5000 IU by an intravenous route at 6 a.m, Intervet, Germany) [19, 20], the control group was injected with only the solvent (normal saline; second group; control), at the same timing. 

### Blood collection and hormonal analysis

Samples (serum and plasma;5mL) were collected from the jugular vein of each jacks and after that, all samples were centrifuged in 2000 rpm/min for a time of 10 minutes and then kept at −20 °C until further hormonal analysis.

Testosterone (T), oestradiol (E2), follicle-stimulating hormone (FSH) and luteinizing hormone (LH) were determined in plasma samples with intra- and inter-assays coefficients of <10 and <14% for all above-mentioned hormones. For measuring the nitric oxide (NO), we collected serum samples, as EDTA could interact with NO [17, 21] as the steps of measuring were previously mentioned using Griess reagent. All samples were collected before injection (hour −1) and at hour 0, then at 1, 2, 4, 6, 8, 12, and 24 hours after injection. Those time points were dependent on other previous studies [19, 20].

### Penile artery (PA) hemodynamics assessment by doppler ultrasound

**Fig. 1:**
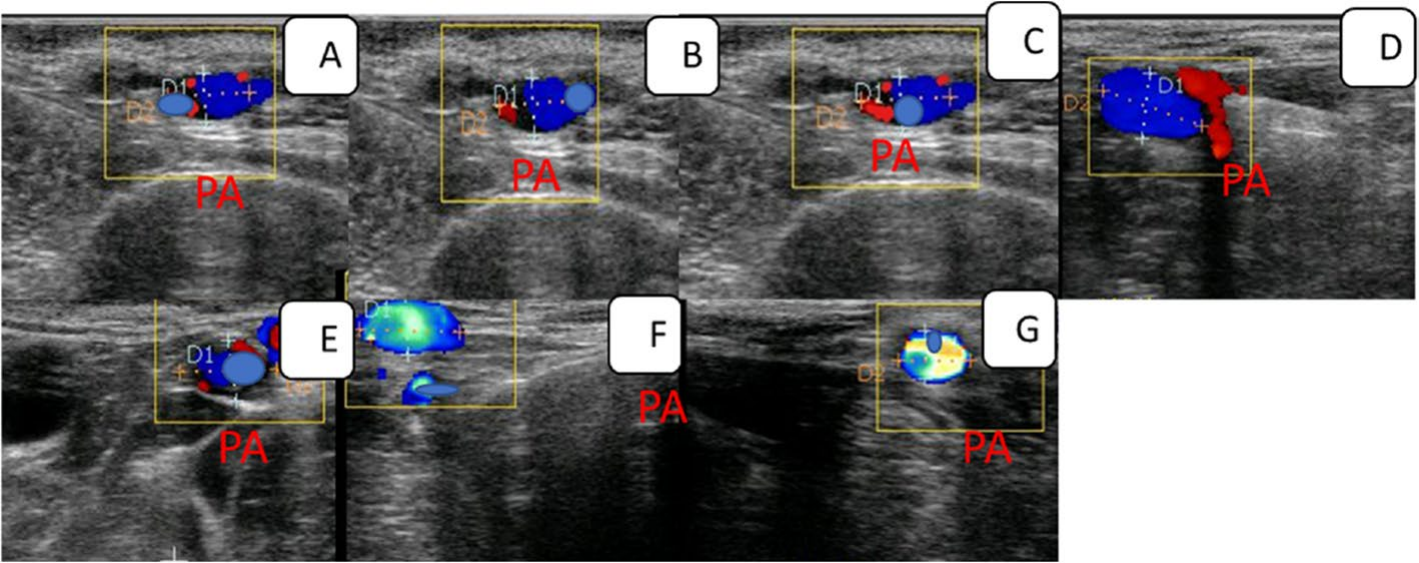
Changes in the penile artery blood flow pattern in the hCG males at hours −1(**A**), 2 (**B**), 4(**C**), 6(**D**), 8(**E**), 12(**F**), and 24(**F**).PA = penile artery

The penile artery blood flow pattern could be estimated by measuring both PA Doppler indices expressed by pulsatility and resistance indices (PI and RI), in addition to penile blood flow volume (mL/min/100g), as all those parameters could be estimated using the spectral mode activation (red and blue colours; Figure 1) tool then spectral mode tool to enter into the penile artery of interest and Make a window gate with 1mm thickness to measure all arterial parameters using Doppler ultrasonography (EXAGO, France) linked with a 5-7.5 MHz linear array probe [22]. The probe was positioned caudally. The penile artery (PA; common penile artery) is a branch from the internal pudendal artery that supplies the penis with adequate blood, along with the scrotal artery that could be reached with the aid of its anatomical consideration [23]. All measurements (three to five) were determined for each parameter calculation to give accurate results. The duration of examination took about 15 min/jacks, and the animal was present in a standing position without any sedating agent.

### Statistical analysis

The results were measured first for the normality check using the Kolmogorov-Smirnov (K-S) test for normality in the SPSS program version 20 to show if the data are homogenous or not. Then all data are expressed as mean ± SEM, the difference between the control and treated group at different known time points in terms of penile hemodynamics pattern(RI, PI, and BFV) and circulating hormones(T, E2, FSH, LH, and NO) and their treatment time interaction were measured using repeated measure ANOVA with general linear model analysis followed by post hoc test. Pearson correlation test was performed to show if there were any interactions between the PA haemodynamic and hormonal levels in jackss. Statistical difference was fixed at *P* <0.05.

## Results

### Anatomical findings

**Fig. 2:**
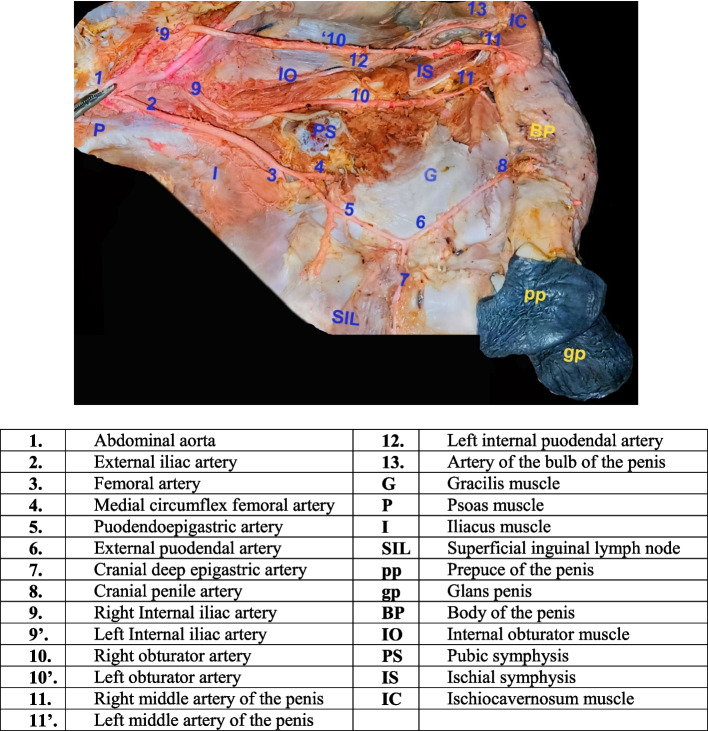
A photograph showing the arterial supply of the penis in the right medial aspect of thigh region of donkey

The arterial supply of the penis of the donkey is achieved via three bilateral main trunks; right and left external pudendal, obturator and internal pudendal arteries; the external pudendal artery (Figure 2) originated from the pudendal-epigastric artery of the external iliac artery then passes obliquely caudoventrally on the lateral aspect of the gracilis muscle terminated with a cranial penile artery that supply the dorsal aspect of the cranial part of penile body and glans penis. The obturator artery (Figure 2) is the thick short artery that detached from the medial aspect of the internal iliac artery and then directs oblique caudoventral on the lateral aspect of the internal obturator muscle and leaves the pelvic cavity by the obturator foramen to pass under the ischial symphysis to supply dorsally the middle part of the corpus cavernosum by the branched tortuous middle artery of the current organ and ischocavernosum muscle. The internal pudendal artery (Figure 2) is considered the pelvic visceral branch of the internal iliac artery, where it passes lateral to the pelvic diaphragm to supply the root of the penis by the artery of the bulb of the donkey penis.

### Main penile artery (PA) blood flow hemodynamics pattern

**Fig. 3:**
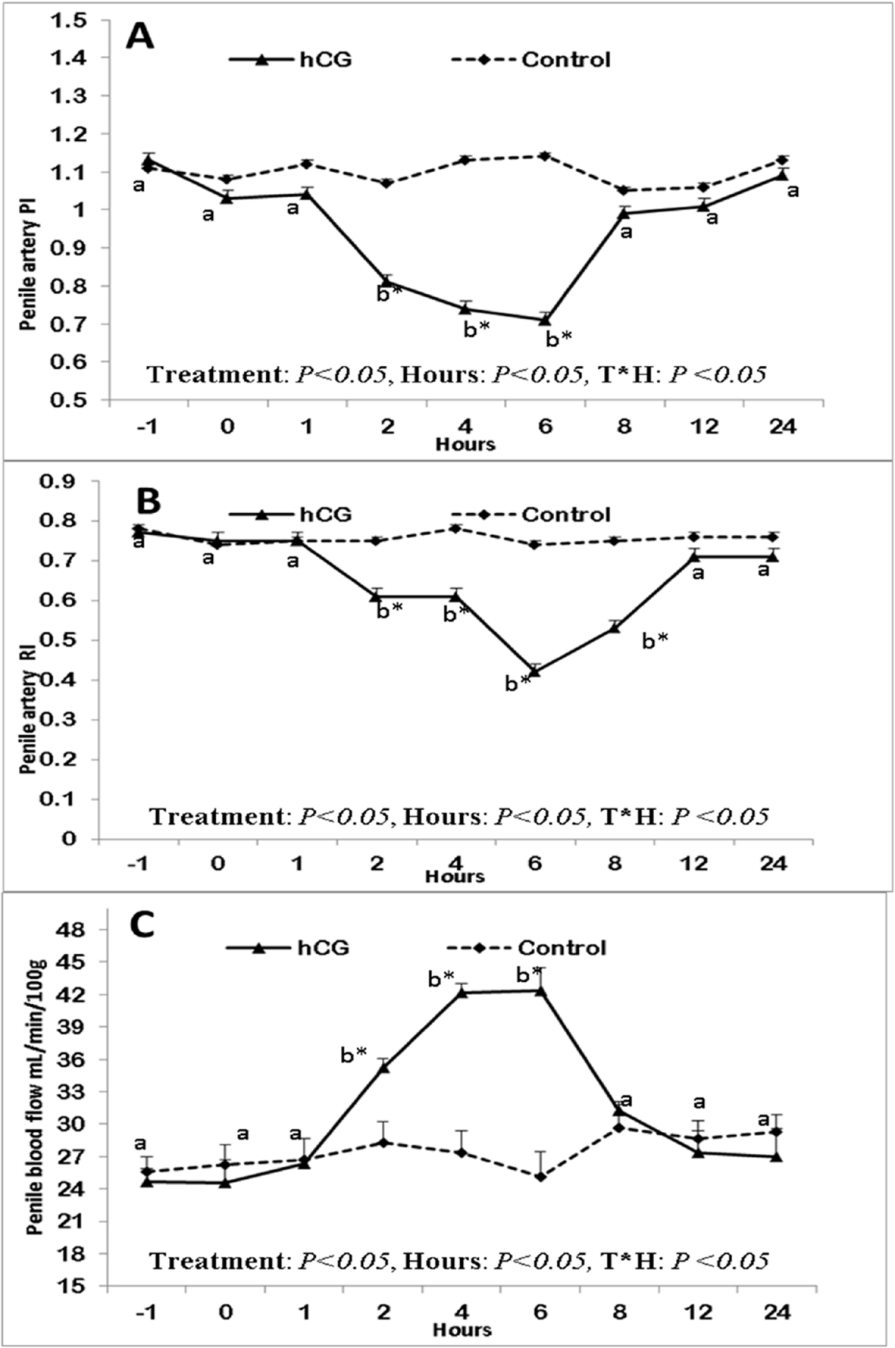
Changes in the penile artery pulsatility index (PI;A),resistance index(RI;B), and penile blood flow volume (mL/min/100g;C) in the treated treated with hCG male donkeys compared to the normal control males. Data obtained as mean ± SEM. ^a and b^ values are significantly different at* P* < 0.05 in the treated group (hCG) along hours of examination, while * value is significantly different at *P* < 0.05 between two groups at the same time point

The penile artery pulsatility index (PI; Figure 3A) and resistance index (RI; Figure 3B) were significantly affected by the treatment (*P*<0.05) and time points (*P*<0.05). both PI and RI declined significantly(*P*<0.05) in the hCG group after a single injection of hCG by 2 hrs, 4 hrs and reached the lowest level at 6 hours then both returned to basal levels until 24 hours after injection compared to the control untreated males. There was a time treatment interaction (*P*<0.05) during the time of observation after injection. The penile blood flow volume ((mL/min/100g; Figure 3C) was affected by the treatment (*P*<0.05), and hours (*P*<0.05). In addition to treatment and time interaction (*P*<0.05), the blood flow volume was elevated significantly and reached the highest level at 6 hours after injection then declined slowly after that.Ten minutes after injection, an erection usually developed, and for one to two hours, the erection and masturbation persisted sporadically. These erections developed as during sexual excitation, leading to the glans penis's eventual engorgement and normal firmness.

### Hormonal alterations

**Fig. 4:**
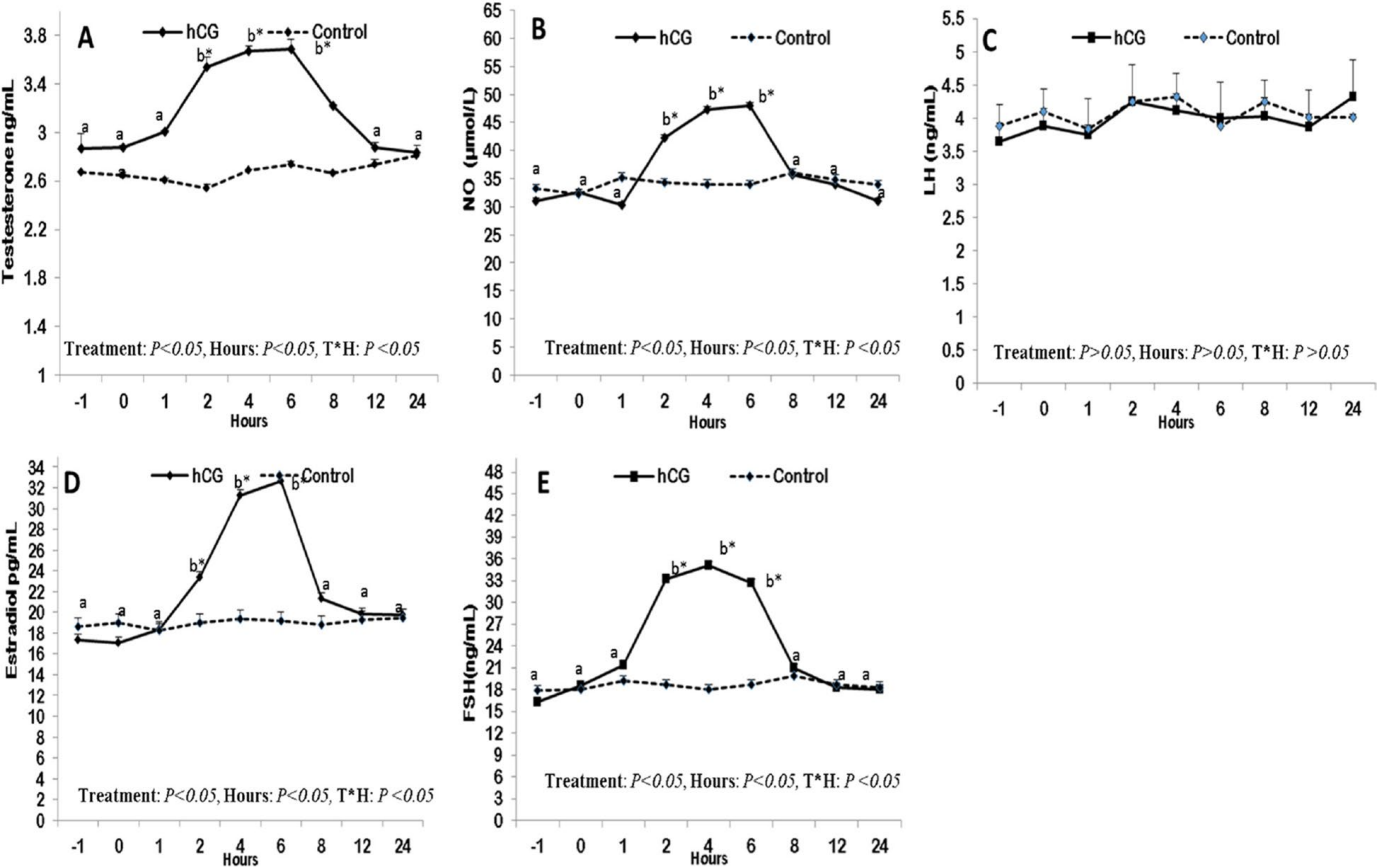
Changes in the levels of testosterone(ng/mL;**A**),nitric oxide (NO;µmol/L;**B**),luteinizing hormone (LH;ng/mL;**C**), Estradiol (pg/mL;**D**),and follicle stimulating hormone(FSH;ng/mL;E) in the treated with hCG male donkeys compared to the normal control males. Data obtained as mean ± SEM. ^a and b^ values are significantly different at* P* < 0.05 in the treated group (hCG) along hours of examination, while * value is significantly different at *P* < 0.05 between two groups at the same time point

Plasma levels of testosterone (ng/mL; Figure 4A), were significantly (P<0.05) changed in the treated Males. A monophasic elevation was reported at 4 and 6 hr. after a single injection compared to the control. Statistically significant changes were determined in nitric oxide levels (NO;µmol/L; Figure 4B) as its level was elevated reaching the peak point 6 hours after injection of hCG, then declined again, but the levels of luteinizing hormone (LH; ng/mL; Figure 4C) did not show any alterations along hour of treatment. The levels of both estradiol (pg/mL; Figure 4D), and follicle-stimulating hormone (FSH; ng/mL; Figure 4E) were elevated reaching the peak point after 6 hrs. of injection for E2 and 4 hours for FSH in addition to the time and treatment interaction that observed in all hormones except LH as depicted in (Figure 4).

### Correlation between penile doppler indices and hormonal levels

In the hCG-treated jacks; the levels of testosterone were negatively correlated with both penile artery Doppler parameters (RI with r = −0.98; *P* = 0.01, and PI with r = −0.77; *P* = 0.01). There was a positive correlation between penile blood flow and both NO and E2 (NO with r=0.66; *P* = 0.01, and E2 with r=0.69; *P* = 0.01), Finally, there was a detected negative relation between penile blood flow and both Doppler indices (PI with r=−0.97;* P* =0.001, and for RI r = −0.98; *P* = 0.001).

## Discussion

Measurements of penile and testicular blood flow in the form of blood flow velocities or both Doppler indices are crucial because, according to Ortiz- Rodriguez *et al.* [24], early detection of genital problems, was linked to a marked vascular disturbance in the form of both blood flow velocities and Doppler indices in both penile and testicular tissues. As a result, Doppler ultrasound is thought to be an effective method for determining testicular blood flow [25, 26].

A review of available literature shows the penile arterial supply of the horse only as the main type in equine species so the special data about the donkey were neglected, so it is the first description to penile artery in donkey, this arterial supply of the donkey's penis is provided by the external pudendal, obturator, and internal pudendal arteries, which are responsible for erection in this current musculocavernous type of penis. These findings align with those reported by [12, 13, 27] in stallions.

Since basal levels of testosterone and gonadotrophins were widely variant among animals, a challenge with hCG is a proper test for investigating the endocrine regulation of equines. hCG is one of the gonadotrophic hormones that induces an elevation of plasma testosterone following the stimulating impulse made on Leydig cells, Parlevliet *et al. *[28] recorded an increase in testosterone concentration within two hours following a single injection of hCG in stallions.

Our results revealed that, four to six hours following hCG injection, a dramatic enhancement of the penile blood supply was achieved, and this enhancement was confirmed by the elevation of testosterone, estradiol and nitric oxide concentrations. These results were parallel to the results recorded by [29] they suggested that hCG treatment could elevate steroid levels throughout the year. Circulating testosterone concentrations were Markedly increased 3 days following hCG injection [29], while in our study this slight increase was noticed 4-6 hours following hCG administration, this difference may be due to species difference or route of hCG administration. Our data were augmented by the results of Doppler indices that are shown in Figure (3).In agreement with our results Pakarinen *et al.* [30] reported variant responses of the steroidal expression in response to hCG stimulation. In agreement with our recorded data, Rana *et al.* [31] found that testosterone concentration was significantly higher 240 minutes following hCG administration in donkeys in comparison with non-injected donkeys.

Erectile function bank on a combination of vascular, structural, endocrine and psychogenic factors. Thus, erectile dysfunction can have a number of either organic or psychogenic factors [32]. The assessment of penile blood flow is critical in determing the efficiency of erection as during the erection [33–35], all known penile arteries were expanded in order to increase the blood flow by filling two tube of the corpus cavernous penis that lead to swelling and make the penile body more longer [36] and therefore the engorgement of the blood lead to enlargement and engorgement of sinusoidal spaces [37]. Therefore, we focused on this study on the Doppler indices to state the amount of penile hemodynamics that directly affects on the erection process as well as testicular hemodynamics [37, 38], as previously know, there was an inverse relationship between both Doppler indices and blood flow rate [39–43].This inverse relationship between Doppler indices and blood flow velocity is related to the calculated equation of resistance and pulsatility indices that are related to peak and end diastolic velocities [44–46].hCG injection activates Leydig cells in animal testes to create testosterone, which is necessary for reproduction, and testeseeone rising [47].

## Conclusion

Due to the production of penile growth through elevation and the improvement of tissue vascularity, temporary penile stimulation by hCG exhibits only minor adverse effects and permits a medical intervention. A significant enhancement of penile blood supply was observed, recorded, and confirmed by the increased levels of testosterone, estradiol, and nitric oxide concentrations following four to six hours post-hCG injection. However, a larger sample size is recommended to standardize the therapeutic and reproductive effects of hCG on jacks.

## Data Availability

All data collected or analyzed during this study are included in this published paper.
